# The Prevalence and Trends of the Early Introduction of Cow Milk to Newborns at Tertiary Care Center: A Risk of Atopy

**DOI:** 10.3390/ijerph18136686

**Published:** 2021-06-22

**Authors:** Ali F. Atwah, Emad A. Koshak, Bakr H. Alhussaini, Saad A. Alsaedi

**Affiliations:** 1 Department of Pediatrics, Faculty of Medicine in Rabigh, King Abdulaziz University, Jeddah 21589, Saudi Arabia; 2 Department of Medicine, Faculty of Medicine in Jeddah, King Abdulaziz University, Jeddah 21589, Saudi Arabia; ekoshak@hotmail.com; 3 Department of Pediatrics, Faculty of Medicine in Jeddah, King Abdulaziz University, Jeddah 21589, Saudi Arabia; Bakrhilal@yahoo.com (B.H.A.); salsaedi@kau.edu.sa (S.A.A.)

**Keywords:** cow milk protein allergy, neonates, exclusive breast feeding, baby-friendly hospital initiative guidelines

## Abstract

Although all health organizations recommend exclusive breastfeeding (EBF), few neonates meet these recommended goals. The early intake of cow milk formulas (CMFs) has been linked to several childhood illnesses, including atopic diseases. Therefore, this study aimed to evaluate the prevalence of early exposure to CMFs in the nursery of a tertiary care hospital in Jeddah, Kingdom of Saudi Arabia. A retrospective review was conducted on the medical records of feeding practices of neonates born in King Abdulaziz University Hospital (KAUH) at Jeddah, Kingdom of Saudi Arabia. Two months from each year (May and December) were selected over the last five years. Approval from the ethical research committee at KAUH was obtained. Eight hundred and ninety-four different neonate files were reviewed. Four hundred and eighty-seven (54.5%) were males. Out of the total of 894, 838 (93.7%) newborns experienced an early introduction to CMFs, 797 (89.1%) received mixed CMF and breast milk, 41 (4.6%) received CMF only, and 56 (6.3%) received exclusive breastfeeding (EBF). Surprisingly, EBF has declined over time, from 39% in May 2016 to 1% in December 2020. The prevalence of early exposure to CMF was very high in newborns at KAUH nursery, and this prevalence was trending upwards. Extensive teaching programs on EBF and allergy prevention for mothers and related health care providers are highly recommended

## 1. Introduction

Atopic disorders are immunoglobulin E (IgE)-mediated diseases that have an increasing prevalence among children and adults and are considered of tremendous concern to community health [1,2,3]. An Australian study has demonstrated the prevalence of atopic disorders to be 32.5% [4], while another study demonstrated a prevalence of 40.3% in Denmark [5]. The prevalence of allergic disorders in Saudi Arabia has been demonstrated to be 41.7% [6].

Cow milk protein allergy (CMPA) is a prevalent disease in pediatrics. The prevalence of CMPA ranges from 0.5% to 3% in persons of 1 year of age who live in developed countries [7]. Saudi’s study has shown a CMPA prevalence of 12.5% and 13% of patients with a suspicion of food allergy [8,9]. Another Saudi study has demonstrated that 27% of patients with food allergies are sensitized to milk [10].

CMFs have largely replaced breast milk due to a lack of societal awareness about the significance of breastfeeding to mothers and babies, the widespread availability of formulas in the market, and the ease of use of such formulas [11].

Globally, the prevalence of EBF of infants (0–5 months) ranged from 28% to 47%. The prevalence in the U.S. was about 47% in the first week of life, 32% at two months, 19% at four months, and 10% at six months [12]. However, the prevalence of EBF in Saudi Arabia was 24.4% at six months of age [13]. EBF was least common among low-birthweight and premature neonates. In addition, mothers of low age, low education or income who smoked during pregnancy are less likely to exclusively breastfeed [12].

Worldwide, the prevalence of exposure to CMF in the nursery is high. In 2000, a cohort study in Finland showed that 87% of the newborns were fed supplementary milk in the maternity hospital [14]. A Chinese study has shown that 50.3% of the mothers exclusively breastfeed their babies before discharge [15]. A study in Vietnam has shown that 33% of mothers had exclusively breastfed their babies before discharge [16]. In Saudi Arabia, almost half of the babies were fed breast milk and formulas in the first month of life [17]. However, no study in Saudi Arabia reports the prevalence of cow milk exposure in the nursery.

The intake of CMFs has been linked to several illnesses in children, including atopy, diabetes mellitus, obesity, and infections [18,19]. Additionally, breast milk intake decreases children’s risk of developing Crohn’s disease [20,21] A few datapoints have suggested that the early introduction of CMFs, especially in the first three days after birth, appears to raise the risk of the development of CMPA, other food allergies, and asthma [22,23].

On the other hand, atopy is less prevalent in breastfeeding infants than in bottle-fed infants [18,24]. The more the infants grow, the more mature their intestines become, decreasing the macromolecules’ permeability and food sensitization [25,26].

Therefore, most scientific societies’ guidelines on infant nutrition that focus on atopic disease prevention recommend EBF in the first four to six months [27,28,29,30]. In addition, such guidelines recommend that all infants should not be fed any complementary food before four months and that all infants should be started on the complementary food by six months [30]. Moreover, the guidelines recommend continuing breastfeeding with complementary food for one year or longer as both mother and baby desire it [31].

WHO and UNICEF recommend the initiation of breastfeeding within the first hour of life and for it to be continued exclusively for six months. They designed the Baby-Friendly Hospital Initiative (BFHI) for successful breastfeeding in 1989. These guidelines consist of ten steps which could improve the rate and duration of EBF. Any hospital that fulfills the criteria is classified as a baby-friendly hospital [32].

Hence, this retrospective study aims to evaluate the prevalence of early exposure to cow milk formulas in the nursery of King Abdulaziz University Hospital and assess compliance to breastfeeding guidelines and recommendations.

## 2. Materials and Methods

This is a retrospective data review-based study that seeks to explore the feeding practices of neonates in the nursery at KAUH in Jeddah, Kingdom of Saudi Arabia.

Approval from the ethical research committee at King Abdulaziz University’s Faculty of Medicine has been obtained (reference number 678-20).

The medical records for healthy newborns who were admitted to the normal nursery in KAUH during May and December over the five years from 2016 to 2020 were reviewed. These two months were chosen because hard copy files were available for those months.

Files were reviewed for neonates’ nationality, gender, gestational age, mode of delivery, birth weight, and the duration that each neonate stayed in the nursery. The nurses wrote all feeding practices data in the feeding charts, including whether breast milk or CMFs were used, feeding frequency, the volume of each feed, and on which days feeding was received in the nursery. Sick and preterm neonates were excluded.

The statistical analysis was performed by IBM-SPSS version 23.0. Kolmogorov–Smirnov tests were used to assess normality for data distribution. The categorized data were expressed in terms of frequency (%), and the parametric data were expressed in terms of medians [25th and 75th interquartile (IQR)] or means, +/− standard deviation (minimum−maximum). The *p* values were calculated using the Pearson chi-square test (*χ*^2^) for the categorical variables and the Kruskal−Wallis test, with non-normal distribution parametric data.

The definitions used in this paper are as follows:

Exclusive breastfeeding: “No other food or drink, not even water, except breast milk (including milk expressed or from a wet nurse) for 6 months of life, but the infant can receive Oral Rehydration Solution, drops, and syrups (vitamins, minerals, and medicines)” [33].

Cow milk formula feeding: Neonates receive formulas only, without any breastfeeding.

Mix: Combined use of breast milk and cow milk formulas.

Late-preterm: Birth at “34(0/7) through 36(6/7) weeks’ gestation” [34].

Low birth weight (LBW): “birth weight of less than 2500 g” [35].

## 3. Results

The total number of neonates was 894; 487 (54.5%) were males, and 407 (45.5%) were females. Six hundred and sixty-two (74%) of the neonates were Saudis, and 232 (26%) neonates were non-Saudis. Six hundred and forty-five newborns (72.1%) were born through spontaneous vaginal delivery, and 249 (27.9%) were born by cesarean section. The mean birth weight was 3.06 ± 0.45 kg with 73 (8.2%) newborns having a birth weight less than 2.5 kg. Twenty-three cases were excluded because of missing data. The other characteristics of all newborns are shown in (Table 1).

**Figure 1:**
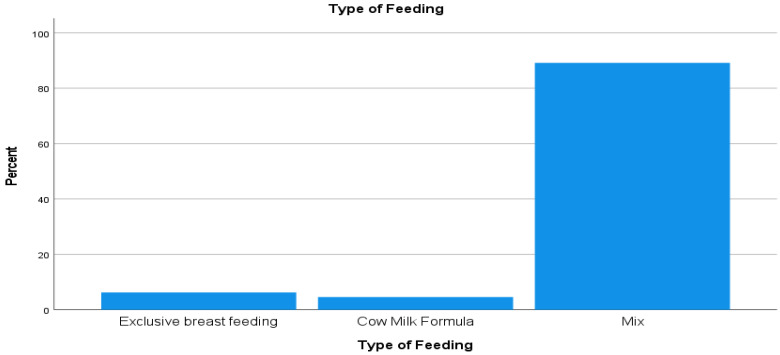
Types of feeding.

The mean duration of nursery stays was 2.3 ± 1.4 days. The prevalence of early introduction to CMFs was 838 (93.7%), and the breakdown was as follows (Figure 1): 797 (89.1%) neonates received a mix of CMFs and breast milk, 41 (4.6%) neonates received CMFs solely, and only 6.3% had received EBF. The total mean and volume of feeds were 6.4 ± 6.1 feeds and 177.4 ± 182.7 mL, respectively. Most newborns (93.1%) received CMFs on the first day of life.

The characteristics of the newborns according to the type of feeding are shown in (Table 2). There was a significant positive relation between EBF and vaginal deliveries (*p* < 0.05). Additionally, there was a nearly significant positive relation between EBF and full-term deliveries (*p* = 0.057) and LBW (*p* = 0.072). There was no significant difference between males and females and no difference between Saudis and non-Saudis.

**Figure 2:**
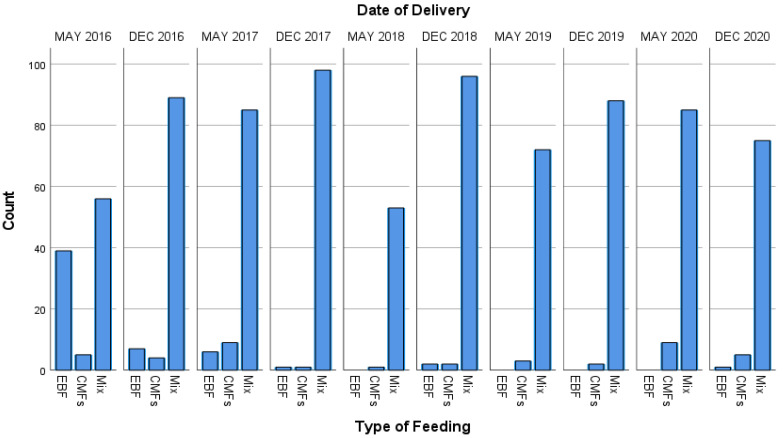
Number of patterns of feeding per month.

The highest prevalence of EBF was 39%, which occurred in May 2016 for unknown reasons. However, exposure to CMFs reached 100% in May 2018, May 2019, December 2019, and May 2020, as shown in (Figure 2).

Surprisingly, the trend of EBF has been declining over the last five years: from 39% in May 2016 to almost 1% in December 2020, as shown in (Table 3). There are no clear reasons for this decline.

## 4. Discussion

This study shows that CMFs were introduced to nearly to all newborns in the nursery of King Abdulaziz University Hospital. This prevalence is close to the findings from other international studies conducted in Finland [14] and Vietnam [16].

Nutrition in early life is crucial to an infant’s growth, neurodevelopment, and physiological health in the future. EBF in the neonatal period protects against developing allergic, metabolic, and infectious diseases in later life [36]. Furthermore, EBF has financial advantages to families and societies. Breast milk reduces the family expenditure on CMFs and decreases the fees and pressure on hospitals from the diseases associated with CMFs [37].

Babies who were delivered as late-preterm or LBW or through cesarean section are less likely to be exclusively breastfed [38,39,40,41]. Preterm infants often do not have sufficient strength to initiate breastfeeding. Breastmilk deficiencies or absences are substantial obstacles in establishing breastfeeding [42]. Mothers are encouraged to start breast milk expression as soon as possible to increase their breast milk volume [43,44]. However, it is difficult for women to move after undergoing a cesarean section, making breastfeeding challenging for mothers [45]. 

Most allergy guidelines recommend EBF for the first four to six months after birth [27,28,29,30]. Finally, the most recent recommendations of EAACI state that supplementation with milk formula in the first week of life should be avoided [46].

All hospitals should follow the WHO’s Ten Steps to Successful Breastfeeding. However, it is not easy to follow these steps in practice because the nursery has a high load of deliveries, the mother lacks the willingness to breastfeed her baby, or the mother finds breastfeeding to be challenging in the first days after delivery.

Many studies have provided strong evidence that the BFHI is effective in increasing the rates of EBF [47,48]. Studies have also suggested that baby-friendly practices increased the duration of breastfeeding [49].

Several factors that influence exclusive breastfeeding in hospitals pertain to the mothers and healthcare providers. The mothers’ decisions and knowledge about the benefit of breast milk are crucial for successful EBF [50,51,52]. In addition, knowledge of breastfeeding skills and family support can help mothers to overcome barriers to breastfeeding [50].

All healthcare providers, lactation consultants, physicians, and midwives, must have the fundamental knowledge and skills needed to help mothers in exclusive breastfeeding [53]. The training of a healthcare provider (especially fresh graduates) is essential to comply with baby-friendly practices and commit to the baby-friendly philosophy [54].

This study’s limitations include its retrospective nature and that it did not include the feeding practices of all healthy neonates admitted to the nursery during the study period. The study included only those neonates who were born during May and December of each year.

## 5. Conclusions

This retrospective study shows that most newborns in the nursery of King Abdulaziz University Hospital received CMFs in the first few days of life. Unfortunately, this is not compatible with the recommendations of most international guidelines for infant nutrition. More effort should be placed on educating mothers about the importance of exclusive breastfeeding. This education should not be limited to education just after delivery but should also include education during child-bearing age and pregnancy.

## Figures and Tables

**Table 1:** Characteristics of all the newborns (*n* = 894).

Characteristics	Values
Nationality	
Saudi	662 (74.0%)
Non-Saudi	232 (26.0%)
Gender	
Male	487 (54.5%)
Female	407 (45.5%)
Gestational age	
Full term	843 (94.3%)
Late pre-term	51 (5.7%)
Mode of delivery	
SVD	645 (72.1%)
Cesarean section	249 (27.9%)
Birth weight (kg)	3.06 ± 0.45 (1.00–4.58)
Low birth weight	73 (8.2%)
Nursery days	2.26 ± 1.4 (1.00–25.00)
Types of feeding	
Exclusively breast milk	56 (6.3%)
Cow milk formula	41 (4.6%)
Mix	797 (89.1%)
Number of feeds	6.42 ± 6.08 (0.00–55.0)
Volume of feeds (ml)	177.40 ± 182.66 (0.00–1635.00)
Days of feeds	
First day	832 (93.1%)
Second day	445 (49.8%)
Third day	187 (20.9%)

**Table 2:** Characteristics of the newborns according to type of feeding.

Characteristics	Exclusive Breast Milk (*n* = 56)	Cow Milk Formula Feeding (*n* = 40)	Mix (*n* = 798)	Significance *(p*-Value)
Nationality				0.88
Saudi (*n* = 662)	41 (6.2%)	10 (1.5%)	611 (92.3%)	
Non-Saudi (*n* = 232)	15 (6.5%)	30 (12.9%)	187 (80.6%)	
Gender				0.49
Male (*n* = 487)	28 (5.7%)	23 (4.7%)	436 (89.5%)	
Female (*n* = 407)	28 (6.9%)	17 (4.2%)	362 (88.9%)	
Gestational age				0.057
Full term (*n* = 843)	56 (6.6%)	38 (4.5%)	749 (88.8%)	
Late preterm (*n* = 51)	0	2 (3.9%)	49 (96.1%)	
Mode of delivery				0.001
SVD (*n* = 645)	53 (8.2%)	36 (5.6%)	556 (86.2%)	
Cesarean section (*n* = 249)	3 (1.2%)	4 (1.6%)	242 (97.2%)	
Birth weight (grams)	3.14 ± 0.38 (2.50–4.20)	3.06 ± 0.48 (2.48–4.24)	3.06 ± 0.45 (1.00–4.58)	0.386
Low birth weight (*n* = 73)	1 (1.4%)	1 (1.4%)	71 (97.2%)	0.072
Median (25th–75th IQR)	3.06 (2.86–3.37)	2.98 (2.68–3.39)	3.06 (2.75–3.06)	
Nursery days	1.61 ± 0.59 (1.00–3.00)	2.32 ± 1.46 (1.00–8.00)	2.30 ± 1.41 (1.00–25.00)	0.001
Median (25th–75th IQR)	2 (1.00–2.00)	2 (2.00–3.00)	2 (2.00–3.00)	
Number of feeds	-	13.5 ± 5.8 (7.00–28.0)	6.45 ± 5.6 (1.0–33.0)	0.001
Median (25th–75th IQR)		12. (9.00–17.50)	4.00 (2.00–9.00)	
Volume of feeds (mL)	-	386.00 ± 207.45 (165–1000)	179.46 ± 175.67 (10–1635)	0.001
Median (25th–75th IQR)		310 (241.25–431.25)	115 (60–245)	

**Table 3:** Trend of feeding according to type of months.

Months	EBF	CMFs	Mix
May 2016	39 (39.0%)	5 (5.0%)	56 (56.0%)
December 2016	7 (7.0%)	4 (4.0%)	89 (89.0%)
May 2017	6 (6.0%)	9 (9.0%)	85 (85.0%)
December 2017	1 (1.0%)	1 (1.0%)	98 (98.0%)
May 2018	0 (0%)	1 (1.9%)	53 (98.1%)
December 2018	2 (2.0%)	2 (2.0%)	96 (96.0%)
May 2019	0 (0%)	3 (4.0%)	72 (96.0%)
December 2019	0 (0%)	1 (1.1%)	89 (98.9%)
May 2020	0 (0%)	9 (9.6%)	85 (90.4%)
December 2020	1 (1.2%)	5 (6.2%)	75 (92.6%)

## Data Availability

Not applicable.
